# Parity, Age at First Birth, and Risk of Death from Asthma: Evidence from a Cohort in Taiwan

**DOI:** 10.3390/ijerph110606147

**Published:** 2014-06-11

**Authors:** Chih-Cheng Chen, Hui-Fen Chiu, Chun-Yuh Yang

**Affiliations:** 1 Department of Pediatrics, College of Medicine, Kaohsiung Chang-Gung Memorial Hospital and Chang-Gung University, Kaohsiung 833, Taiwan; E-Mail: charllysc@hotmail.com; 2 Department of Pharmacology, College of Medicine, Kaohsiung Medical University, Kaohsiung 807, Taiwan; E-Mail: chiu358@yahoo.com.tw; 3 Department of Public Health, College of Health Sciences, Kaohsiung Medical University, Kaohsiung 807, Taiwan; 4 Division of Environmental Health and Occupational Medicine, National Health Research Institute, Miaoli 350, Taiwan

**Keywords:** asthma, parity, mortality, cohort study, Taiwan

## Abstract

This study was undertaken to examine whether there is an association between age at first birth and parity and risk of asthma death. The study cohort consisted of 1,292,462 women in Taiwan who had a first live birth between 1 January 1978 and 31 December 1987. We tracked each woman from the date of their first childbirth to 31 December 2009, and their vital status was ascertained by linking records with the computerized mortality database. Cox proportional hazard regression models were used to estimate hazard ratios of death from asthma associated with parity and age at first birth. A trend of increasing risk of asthma death was seen with increasing age at first birth. The adjusted hazard ratio was 0.75 (95% confidence interval (CI) = 0.53–1.08) among women with two live births and 0.53 (95% CI = 0.36–0.78) among those with three or more births, compared with women who had one live birth. There was a significant decreasing trend in adjusted hazard ratios of asthma death with increasing parity. This study is the first to provide evidences to support an association between reproductive factors (parity and early age at first birth) and the risk of asthma death.

## 1. Introduction

Asthma prevalence and severity are higher among boys than girls before puberty, about the same in both sexes during teenage years, and thereafter asthma predominates among females [1,2]. This pattern continues until menopause, when asthma incidence among women decreases [3] to become equal to that of men [2]. In addition, asthma severity has been shown to vary during the menstrual cycle and pregnancy [4]. These findings suggest that reproductive hormones influence the development of asthma and asthma severity [5].

Several mechanisms have been proposed to explain the role of female hormones in asthma risk. Several animal studies have reported increased susceptibility to allergic airway disease in female mice compared to male mice. Estrogens appear to exert most of their effects through estrogen receptors alpha and beta, both of which are present in the lung [6]. Sex hormone concentrations increase markedly after puberty. Women with early menarche have higher estrogen levels and are exposed to a greater cumulative estrogen and progesterone concentrations than women with later onset of menarche [7]. Estrogens has been shown to activate endothelial nitric oxide synthase [8,9]. Nitric oxide has been implicated in the pathogenesis of asthma [10] and levels vary with the menstrual cycle [11].

To our knowledge, there is only one study that investigated a possible link between age at first birth and parity and the risk of asthma. Jenkins *et al.* reported that the risk of later-onset asthma among women who had no asthma by age seven was associated with increased parity. However, the risk of asthma decreased with increasing age at first birth [12].

We have now studied a cohort of women who experienced a first and singleton childbirth between 1 January 1978 and 31 December 1987 to explore further the association between age at first birth and parity and the risk of asthma death in Taiwan.

## 2. Experimental Section

### 2.1. Data Source

Registration of births is required by law in Taiwan. It is the responsibility of the parents or the family to register infant births at a local household registration office within 15 days. The Birth Registration System, which is managed by the Department of Interior, released computerized data on live births since 1978. The registration form, which requests information on maternal age, education, parity, gestational age, date of delivery, infant gender, and birth weight, is completed by the physician attending the delivery. Because most deliveries in Taiwan take place in either a hospital or clinic and the birth certificates are completed by physicians attending the delivery and it is mandatory to register all live births at local household registration offices; therefore the birth registration data are considered complete, reliable and accurate [13,14].

### 2.2. Study Population

The study cohort consisted of all women with a record of a first and singleton childbirth in the Birth Register between 1 January 1978 and 31 December 1987. There were 1,292,462 first and singleton births occurred in Taiwan between 1978–1987. Information on any subsequent births was also retrieved from the Birth Register.

### 2.3. Follow-up

Each woman has her own unique personal identification number. Using this number, we tracked each woman from the time of their first childbirth to 31 December 2009, and their vital status was ascertained by linking records with the computerized mortality database, identifying the date of any deaths occurring in this cohort. Since it is mandatory to register death certificates at local household registration offices, the mortality statistics in Taiwan were considered to be highly accurate and complete [13,14].

### 2.4. Statistics

The person-years of follow-up for each woman was calculated from the date of first childbirth to the date of death or 31 December 2009. Death rates were calculated by dividing the number of deaths from asthma by the number of person-years of follow-up. Cox proportional hazard regression models were used to estimate hazard ratios (HRs) of death from asthma associated with parity (the number of children recorded in the last childbirth record of each woman registered during follow-up) and age at first childbirth. The 95% confidence intervals (CIs) for HRs were also calculated. Asthma is defined according to the International Classification of Disease, Injury, and Causes of Death (9th revision) (ICD code 493). The variables in the final model included age at first childbirth (<25, 26–30, >30 years), parity (one, two, three or more), marital status (married, unmarried), years of schooling (<9, >9 years), and birth place (hospital/clinic, home). The above mentioned variables were included in the final model because they were potential confounders. The proportional hazards assumption was assessed for all above-mentioned variables, and no violations were observed. Analyses were performed using the SAS statistical package (version 9.1, SAS Institute Inc., Cary, NC, USA). All statistical tests were two-sided; *p* values of less than 0.05 were considered to be statistically significant.

## 3. Results and Discussion

Altogether 1,292,462 primiparous women with complete information were included in the analysis. A total of 34,980,246 person-years were observed during the follow-up period from the time of their first childbirth to 31 December 2009. There were 237 asthma deaths, yielding a mortality rate of 0.68 cases per 100,000 person-years.

Table 1 gives the numbers of person-years of follow-up and asthma deaths by age at recruitment (age at first birth), parity, marital status, years of schooling, and birth place. The mortality rate was 1.17 among women who had given birth to one child, 0.69 among those who had had two children, and 0.54 among those who had given birth to 3 or more children.

**Table 1:** Demographic characteristics of the study cohort.

Parameters	Variables	No. of Subjects	Follow-up Person-Years	No. of Deaths from Asthma	Mortality Rate (Per 100,000 Person-Years)
Age at recruitment (1st birth)	≤25	859,942	23,576,614.50	137	0.58
	26–30	372,895	9,873,247.00	70	0.71
	>30	59,625	1,530,384.50	30	1.96
Parity	1	157,207	4,170,772.33	49	1.17
	2	564,727	15,124,112.33	104	0.69
	≥3	570,528	15,685,361.33	84	0.54
Marital status	Married	1,260,615	34,115,479.25	222	0.65
	Not married	31,847	864,766.75	15	1.73
Years of schooling	≤9	722,518	19,850,938.17	167	0.84
	>9	569,944	15,129,307.83	70	0.46
Birth place	Hospital/clinic	1,245,925	33,638,862.83	218	0.65
	Home	46,537	1,341,383.17	19	1.42

The multivariate-adjusted hazard ratios and 95% CIs are shown in Table 2. An older age at first birth was associated with an increased risk of asthma death. The adjusted hazard ratio was 1.14 (95% CI = 1.05–1.91) for women who gave birth between 26 and 30, and 3.15 (95% CI = 2.06–4.82) for women who gave birth after 30 years of age, respectively, when compared with women who gave birth before age 25. A trend of increasing risk of asthma death was seen with increasing age at first birth (*p* for trend <0.0001).

**Table 2:** Association between parity, age at first birth, and relative risk of death from asthma over a 32-year follow-up period.

Parameters	Variables	Crude RR (95% CI)	Multivariate-adjusted RR * (95% CI)
Age at recruitment (1st birth)	≤25	1.00	1.00
	26–30	1.25 (0.93–1.66)	1.14 (1.05–1.91)
	>30	3.50 (2.36–5.20)	3.15 (2.06–4.82)
		*p < 0.0001 for linear trend*	*p < 0.0001 for linear trend*
Parity	1	1.00	1.00
	2	0.58 (0.42–0.82)	0.75 (0.53–1.08)
	≥3	0.45 (0.32–0.34)	0.53 (0.36–0.78)
		*p < 0.0001 for linear trend*	*p = 0.0009 for linear trend*
Marital status	Married	1.00	1.00
	Not married	2.66 (1.58–4.48)	1.94 (1.14–3.31)
Years of schooling	≤9	1.00	1.00
	>9	0.56 (0.42–0.74)	0.48 (0.36–0.65)
Birth place	Hospital/clinic	1.00	1.00
	Home	2.12 (1.33–3.40)	2.05 (1.27–3.31)

After adjustment for age at first birth, marital status, years of schooling, and birth place, the adjusted hazard ratio was 0.75 (95% CI = 0.53–1.08) among women with two live births and 0.53 (95% CI = 0.36–0.78) among those with three or more births, compared with women who had given birth to only one child. There was a statistically significant decreasing trend in the adjusted hazard ratio for asthma death with increasing parity (*p* for trend < 0.0001).

To our knowledge, this is the first prospective study to examine the relationship between reproductive factors (parity and age at first birth) and the risk of asthma death. Previous study used cross-sectional design to determine the relationship between a woman’s reproductive history and adult-onset asthma [12]. In this prospective cohort study, we found that there is a significant protective effect of parity on the subsequent risk of death from asthma. Our finding of a reduced risk of death from asthma associated with higher parity is not in agreement with a previous study conducted in Australia, which reported an increased risk of later-onset asthma associated with higher parity [12].

The mechanism by which increased parity may confer protection against the future development of asthma in women remains unclear. Regulatory T cells (Tregs) have been shown to play an important role in moderating allergic asthma [15] and may be involved in the increased susceptibility of female mice for asthma [16]. Female mice have fewer Tregs and therefore have less protection against inflammatory stimuli such as allergens [16]. There is experimental evidence that estrogens have an antiinflammatory effects. In mice, *in vivo* administration of estrogen in pregnancy-like concentrations was found to expand the pool of Tregs and to enhance their antiinflammatory function [17]. Pregnancy elevates serum estrogen levels about 100 fold [18]. Increasing parity is associated with an overall increase in lifetime exposure of sex hormones (including estrogen). Thus, if estrogens are associated with a reduced risk of death from asthma, we would expect pregnancy to offer some protection from asthma death. Our data provide support for this hypothesis.

Contrary to a previous study [12], in the present prospective cohort study, we found that risk of death from asthma increased with increasing age at first birth after adjusting for parity. To our knowledge, this is the first study to find a significant positive association between age at first birth and risk of death from asthma. The reasons are unknown. It may be that an earlier age at first birth played a protective role in asthma risk via elevated levels of some hormones (including estrogens) during pregnancy. Moreover, it has been found that age at menarche (and thus exposure to period estrogens stimulation) is related to age at which a woman delivers her first child [19]. Experimental evidence pointing to estrogens as suppressors of allergic airway inflammation by increasing the number of Tregs suggests that estrogens could be the crucial hormone responsible for the positive association between age at first birth and risk of asthma death [17]. However, because there is no evidence to date for an association between age at first birth and risk of death from asthma, the possibility that this is a chance finding also needs to be considered. Clearly, more work will be needed before the influence of age at first birth on the risk of death from asthma is understood.

The completeness and accuracy of the death registration system should be evaluated before any conclusion based on the mortality analysis is made. In the event of a death in Taiwan, the decedent’s family is required to obtain a death certificate from the hospital or local community clinic, which then must be submitted to the household registration office in order to cancel the decedent’s household registration. The death certificate is required in order to have the decedent’s body buried or cremated. Death certificates must be completed by physicians in Taiwan. It is also mandatory to register all deaths at local household registration offices, the death registration is accurate, reliable and complete. The complete population coverage and follow-up made possible by the national identification number has left the study without selection bias. Information bias is also unlikely to be important for parity and age at first birth.

Taiwan inaugurated its National Health Insurance (NHI) program in 1995 to finance healthcare for all citizens of Taiwan. As of 2007, the NHI program provided coverage for 22.60 million out of 22.96 million Taiwanese (coverage rate 98.4%) [20]. Taiwan is a small island with a convenient communication network. It is believed that all asthma cases had access to medical care. Furthermore, it seems unlikely that medical utilization would be correlated with parity. We therefore believe that medical utilization should not have a substantive effect on the associations we observed.

Mortality data rather than data on inpatient cases was used to assess the association between parity, age at first birth, and risk of death from asthma in this study. The mortality of a disease is a function of its incidence and fatality. No previous study has investigated the differences in survival rates of asthma between high and low parity. Thus, this is a limitation that should be considered.

Our data takes into account the effect of the number of children on the risk of mortality from asthma. The risk progressively declines with each additional birth. The birth registration system in Taiwan covers only live births. We were unable to examine the possible role of nulliparity on the risk of asthma death. The generalizability of our findings is thus limited. Further study in independent cohorts with the inclusion of non-parous women for follow-up studies of the association between parity and the risk of asthma death are needed.

Selection effects are also likely to be an important explanation for this association. Women with severe asthma in their reproductive years may affect opportunities for marriage, motherhood, age of the first childbirth and decisions about subsequent childbearing. We do not know how asthma status may have affected a woman’s decision to have more children. If women with severe asthma opted to have no more children or may have had reduced fertility as a result of their severe asthma status, our results may be biased.

Of the studies that have evaluated the use of oral contraceptives (OCs) and the risk of developing asthma, one study reported a decreased risk [12], two studies showed an increased risk [21,22], and one has failed to find statistically significant association [23]. Regarding menopausal hormone therapy (MHT), previous studies have largely reported a positive association between the use of MHT and asthma risk. Three cross-sectional studies have reported an association between prevalent asthma and asthma-like symptoms and the use of MHT in women [23,24,25] and three prospective studies have suggested that MHT use was associated with an increased risk of newly diagnoses of asthma [3,5,26].

We were unable to adjust for these two hormonal factors in the current study due to the lack of available data. Since the use of OCs and MHT are low in Taiwan compared with Western countries [27,28], the confounding effect resulting from these two factors should be small, if existed at all. Furthermore, if the association between these two potential confounding variables and the risk of asthma death is not as strong as the one that has been observed for parity and age at first birth, adjustment of these variables will not qualitatively change the conclusion.

Breastfeeding causes hormonal changes, possibly reduced estrogen and increased prolactin production, which inhibits the initiation or growth of breast cancer [29,30,31]. This phenomenon (low-estrogen environment) possibly could increase the risk of asthma death if the hypothesis that estrogen exposure confer a protective effect on the risk of asthma death is correct. No previous study has investigated the impact of lactation on the risk of asthma. Data were also not available in this study to adjust for the effect of this factor for asthma death. However, additional studies are needed to evaluate the effect of breastfeeding on asthma risk especially among premenopausal women and explore further the role of estrogen in the etiology of asthma. Furthermore, length of breastfeeding as well as the number of children is often considered dependent on socioeconomic conditions. There is no information available on income levels for the study subjects. However, years of schooling was used as a proxy for socioeconomic status and this factor was adjusted for in the multiple regression analysis.

## 4. Conclusions

In summary, we found that there was a trend for increasing parity to be associated with decreasing risk for asthma death. In addition, we found that risk of asthma death increased with increasing age at first birth. This study is the first to suggest that reproductive factors (parity and early age at first birth) may confer a protective effect on the risk of asthma death.
